# Chronic obstructive pulmonary disease and atherosclerosis: common mechanisms and novel therapeutics

**DOI:** 10.1042/CS20210835

**Published:** 2022-03-23

**Authors:** Kurt Brassington, Stavros Selemidis, Steven Bozinovski, Ross Vlahos

**Affiliations:** School of Health and Biomedical Sciences, RMIT University, Bundoora, VIC 3083, Australia

**Keywords:** atherosclerosis, cardiovascular disease, chronic obstructive pulmonary disease, cigarette smoke, lung inflammation, oxidative stress

## Abstract

Chronic obstructive pulmonary disease (COPD) and atherosclerosis are chronic irreversible diseases, that share a number of common causative factors including cigarette smoking. Atherosclerosis drastically impairs blood flow and oxygen availability to tissues, leading to life-threatening outcomes including myocardial infarction (MI) and stroke. Patients with COPD are most likely to die as a result of a cardiovascular event, with 30% of all COPD-related deaths being attributed to cardiovascular disease (CVD). Both atherosclerosis and COPD involve significant local (i.e. lung, vasculature) and systemic inflammation and oxidative stress, of which current pharmacological treatments have limited efficacy, hence the urgency for the development of novel life-saving therapeutics. Currently these diseases must be treated individually, with no therapies available that can effectively reduce the likelihood of comorbid CVD other than cessation of cigarette smoking. In this review, the important mechanisms that drive atherosclerosis and CVD in people with COPD are explained and we propose that modulation of both the oxidative stress and the inflammatory burden will provide a novel therapeutic strategy to treat both the pulmonary and systemic manifestations related to these diseases.

## Introduction

Chronic obstructive pulmonary disease (COPD) is a heterogeneous disease that is currently the third leading cause of deaths globally [1,2]. This debilitating, irreversible disease is attributed to a persistent airflow limitation and overstated pulmonary inflammatory response, resulting from exposure to noxious gases and particles [3–5]. COPD is responsible for ∼3 million deaths and an overwhelming €82 billion in global healthcare costs annually [1,6]. The huge financial burden associated with this disease is largely due to the management of its comorbidities, other underlying medical conditions such as cardiovascular disease (CVD), and acute exacerbations of COPD (AECOPD) which are defined as ‘an acute worsening of respiratory symptoms that result in additional therapy’ [7]. It is established that comorbid CVD is a key contributor to the morbidity and mortality associated with COPD, with approximately 30% of all COPD patients dying as a result of cardiovascular manifestations [8]. Cigarette smoking accounts for 95% of all cases of COPD in industrialized countries, however airborne pollutants and exposure to noxious gasses may also lead to the onset or development of this disease [3,9,10]. COPD is a chronic inflammatory disease of the lungs, and this inflammatory burden promotes structural remodeling and damage to the small airways, large airways and lung parenchyma; impairing the elastic recoil of the lung, and ultimately reducing lung function [4,11–13]. It is this persistent pulmonary inflammation and increased oxidative stress from exposure to CS that is believed to drive atherosclerosis and CVD in COPD patients. There is evidence to suggest that pro-inflammatory mediators and reactive oxygen species (ROS) spill over into the systemic circulation, driving extrapulmonary pathologies. Studies have shown that CS promotes both pulmonary and systemic inflammation, systemic oxidative stress, pulmonary endothelial dysfunction and enhanced levels of circulating pro-coagulant mediators [14–16]. Exposure to CS exerts deleterious systemic effects that contribute to the development of chronic comorbid diseases and further functional impairments which reduce the overall quality-of-life of these patients.

Reduced lung function has been implicated in cardiovascular mortality in patients with COPD, with a study concluding that a decline in forced expiratory volume in 1 s (FEV_1_) is strongly correlated with increased cardiovascular mortality [17]. This reduced lung function is the result of airway remodeling due to repeated cycles of injury and repair of the extracellular matrix (ECM) promoting dysregulated deposition of ECM proteins, enhancing airway stiffness [17,18]. The mechanisms linking these COPD-induced pulmonary and systemic manifestations are largely unknown, however it is clear that these conditions act synergistically, as COPD is the largest driver of pulmonary hypertension, which has been linked to an increase in the likelihood of various cardiovascular complications including chronic heart failure and myocardial infarction (MI) [19]. Pulmonary hypertension is the result of an increase in blood pressure (BP) due to narrowing of the vessel lumen, inflammatory cell recruitment and excessive proliferation of vascular smooth muscle cells (VSMCs), endothelial cells (ECs) and fibroblasts within the vascular wall [20,21]. The inflammatory burden within the lung due to both pulmonary hypertension and COPD puts these patients at an increased risk of developing atherosclerosis and other extrapulmonary comorbidities.

Atherosclerosis is currently the leading cause of stroke, peripheral vascular disease (PVD) and coronary heart disease [22,23]. Various other cardiovascular conditions have also been reported in greater incidence in COPD patients than the healthy population, these include: PVD, cardiac arrhythmias, congestive heart failure and coronary artery disease, with persistent low-grade systemic inflammation being a common link between both CVD and COPD [24,25]. In this review, the deleterious role of pulmonary inflammation and oxidative stress in atherosclerosis will be explored, and we will outline potential novel therapeutic strategies to combat comorbid atherosclerosis and reduce its morbidity and mortality in COPD patients.

## Pathogenesis of COPD

COPD is an incapacitating, irreversible disease of the lungs that leads to emphysema, narrowing of the small airways, persistent pulmonary inflammation and fibrosis [26]. Diagnosis of COPD requires quantification of airflow limitation by either bronchodilator tests or spirometric analysis, where patients with COPD display declined pulmonary function as defined by FEV_1_/forced vital capacity (FEV_1_/FVC) ratio of less than 0.7 [27]. Hallmark features of COPD include chronic cough, breathlessness, increased sputum production bronchial inflammation/mucous plugging (obstruction of airways) and dyspnea which have unfavorable effects on the overall quality of life of these patients, which is further worsened by virus and bacteria-induced AECOPD [10]. It is well established that CS promotes pulmonary inflammation, due to enhanced immune cell recruitment to the lungs [28,29]. Recruited innate immune cells include macrophages, neutrophils and eosinophils, stimulate the release of inflammatory cytokines, matrix metalloproteinases (MMPs) and chemotactic proteins [28,30–33]. Macrophages, respond to both endogenous and exogenous stimuli and significantly increase proteinase activity leading to tissue degradation and injury, acting as a first line of defense within the lungs. Fibroblasts and airway epithelial cells also play a crucial role in the secretion of MMPs, however, this is at a much higher concentration in COPD patients than in healthy individuals, hence worsened pulmonary pathologies [34]. Neutrophilic inflammation is a key driver of lung tissue damage in COPD, due to neutrophils having the ability to secrete proteases at high concentrations, with direct correlations being made between increased lung neutrophil number and impaired lung function [35]. A study by Lapperre et al. utilized single-breath N_2_ test (sbN_2_-test) to assess small airway pathology in COPD patients [36]. The study showed that an uneven ventilation of the lung is a direct reflection of airway closure and tissue damage. Bronchial biopsies were also taken to investigate immune cell populations and sputum content in this cohort. The study concluded that an increase in N_2_ concentration was proportional to an increase in subepithelial neutrophil numbers, however this was not observed in any other immune cell type, therefore the sbN_2_-test is an accurate indicator of neutrophilic inflammation-mediated small airway dysfunction in COPD, highlighting the significant role of neutrophils in the development of COPD and pulmonary dysfunction.

Eosinophilic inflammation is often associated with asthma rather than COPD, however emerging evidence suggests that eosinophils may play a crucial role in mediating cell injury as a result of interleukin (IL)-33 and IL-13-induced inflammation [37]. Both blood and sputum eosinophil number is advantageous clinically as it is often used as an indicator of inhaled corticosteroid (ICS) efficacy, as the effect of ICS is greater in patients with eosinophilic airway inflammation [38]. A randomized controlled trial by Siva et al*.* highlighted that through minimizing eosinophilic airway inflammation there is a significant reduction in exacerbation instance and severity [39,40], highlighting the importance of targeted therapeutics in the treatment of COPD. Eosinophils become activated upon exposure to pro-inflammatory mediators such as IL-3 and IL-5, promoting migration to sites of inflammation (i.e. the lungs upon contact with virus) [37,41–44]. These recruited eosinophils then mediate the release of pro-inflammatory mediators such as cytokines and growth factors, which induce persistent lung inflammation and worsened exacerbation severity [45,46]. It is believed that the spillover of these inflammatory mediators, resulting from this exuberant immune cell recruitment occurring in the lungs drives extrapulmonary comorbidities such as CVD, therefore a clear understanding of the mechanisms underlying lung inflammation and the pathogenesis of COPD is crucial to defining these complex comorbid diseases.

## Blood vessel homeostasis

Under normal conditions, the vasculature plays a crucial role in the regulation of blood pressure and flow. The vascular endothelium plays a pivotal role in this fine regulation, as hemodynamic forces exerted on the luminal vascular surface (ECs) because of blood flow/sheer stress, promote endothelial signaling, which maintains homeostatic balance, BP and vascular resistance. This vascular tone homeostasis is the result of complex endothelial regulation and signal transduction from the apical domain of the vascular lumen to the more superficial smooth muscle layer and is achieved through interactions between the endothelial cytoskeletal framework, which comprises microtubules, microfilaments and intermediate filaments [47]. The shear stress exerted on the lumen wall promotes vasodilation through the activation of mechanosensitive ion channels, which trigger a rapid influx of Ca^2+^ into the EC cytoplasm, triggering sheer stress-dependent calcium ion channels. Upon calcium influx, there is a sharp increase in the activity of the endothelial nitric oxide synthase (eNOS) enzyme, which promotes vasodilatory nitric oxide (NO) signal transduction through myoendothelial gap junctions to the underlying VSMCs [2,10,47].

**Figure 1:**
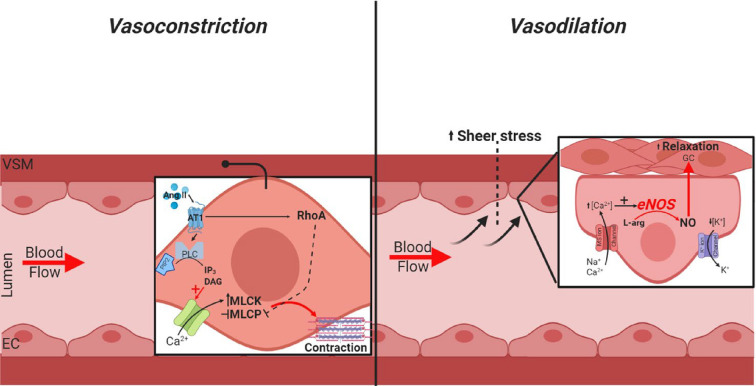
Flow-mediated vasoconstrictive and vasodilatory mechanisms Luminal shear stress promotes Ang II-mediated vasoconstriction through the binding and activation of the Ang II–AT1 G-protein coupled receptor complex, inhibiting the sarcomere phosphatase brake of MLCP and enhancing MLCK activity, promoting smooth muscle-dependent vasoconstriction. Shear stress may also induce vasodilation through EC-mediated NO production and release, driving the activity of guanylyl cyclase within the VSMCs and thus endothelial-dependent vasodilation occurring.

Hemodynamic forces and flow-induced shear stress can also modulate the synthesis of vasoconstrictive factors such as angiotensin II (Ang II). Angiotensin-1 receptor (AT1) stimulation by Ang II promotes G-protein activation of phospholipase C (PLC) causing the hydrolytic conversion of phosphatidylinositiol-4,5-biphosphate (PIP2) into diacylglycerol (DAG) and inositol-1-,4,5-tiphopshate (IP_3_) (Figure 1) [48]. This mediates the opening of cell surface calcium channels as well as the release of Ca^2+^ from intracellular stores. The sudden spike in available Ca^2+^ triggers the activation of myosin light chain kinase (MLCK), promoting phosphorylation and inhibition of myosin light chain phosphatase (MLCP) and subsequent vasoconstriction due to increased myofilament tension. The Ras homolog family member A (RhoA)/Rho-kinase pathway also plays a key regulatory role on vasoconstriction as it acts as a cellular brake, preventing MLCP activity [49,50]. Similarly, the protein kinase C pathway acts by MLCP inhibition via protein phosphatase 1 regulatory subunit 14A (CPI-17) [51,52]. However, a study by Lassѐgue et al. defined a novel role of phosphatidylcholine (PC) in Ang II-mediated smooth muscle contraction. Mechanistically, Ang II activates phospholipase D (PLD) promoting hydrolysis of PC [53]. This PC is further converted into phosphatidic acid and choline, where the phosphatidic acid is then converted into DAG which then goes on to activate the PKC pathway, promoting vasoconstriction and in increase in BP, typical of Ang II stimulation. With a clear understanding of the homeostatic regulators within the vasculature, and how these mechanisms are altered during diseases, such as atherosclerosis where the balance is shifted in favor of vasoconstriction rather than vasodilation, we can potentially develop novel life-saving therapeutics, preventing this dysregulation and reducing the likelihood of cardiovascular manifestations. In the context of comorbid CVD in COPD, damage to the vascular endothelium is believed to mediate the onset of disease, which we found is the result of enhanced oxidative stress and inflammation associated with COPD and CS, putting these patients at a heightened risk of illnesses including atherosclerosis, MI and stroke.

There is emerging interest into perivascular adipose tissue (PVAT) and its ability to influence vascular homeostasis, as it has been shown to contribute directly to vascular function. The PVAT is rich in stromal cells and adipocytes, both of which have the potential to induce paracrine signaling on VSMCs, ECs and induce both ROS and cytokine production upon vascular stimuli such as localized inflammation [54]. Like that observed in atherosclerosis, PVAT dysfunction is largely the result of oxidative stress, hypoxia and inflammation due to immune cell invasion and T-cell recruitment into the PVAT layer [55–57]. There are numerous hypotheses stating that the PVAT may play a crucial role in atherogenesis, which sees activated PVAT causing a subsequent release of proinflammatory and chemotactic mediators. It is believed that the secretion of these factors leads to enhanced immune cell activation and infiltration, plaque formation and endothelial dysfunction [58]. A study by Spiroglou et al. showed that the expression of adiponectin, visfatin and chemerin expression in human periaortic and pericoronary adipose tissues was increased significantly in patients with both aortic and coronary atherosclerosis [59]. These findings suggest that the adipokines; the protein products secreted from the PVAT and immune cell infiltration are directly associated with paracrine signaling and the pathogenesis of atherosclerosis and CVD.

## The detrimental role of COPD in the pathogenesis of atherosclerosis and CVD

**Figure 2:**
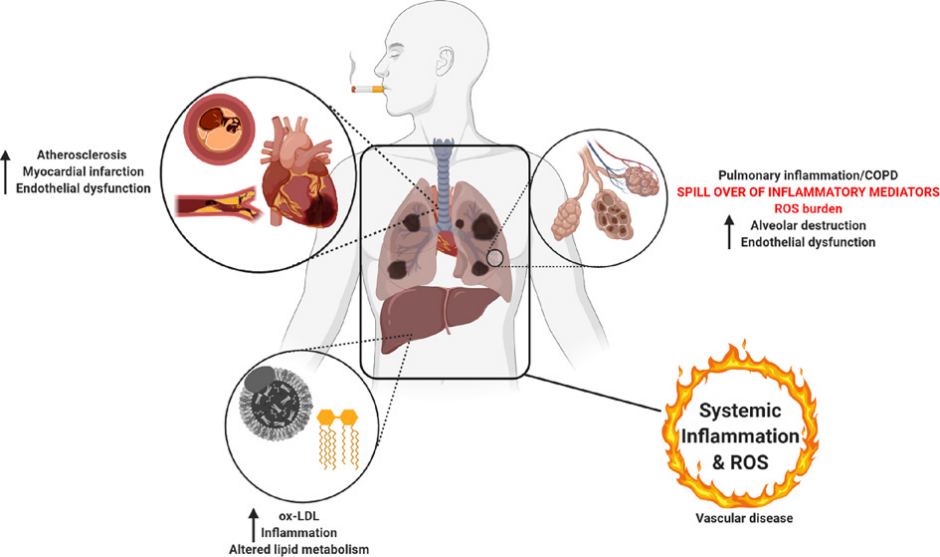
The role of CS and COPD in atherogenesis Exposure to CS promotes pulmonary inflammation and COPD pathogenesis due to over-exuberant ROS and immune cell recruitment into the lungs. These harmful inflammatory mediators and ROS can then spill into the blood leading to a systemic inflammatory response driving various extrapulmonary pathologies including atherosclerosis, MI as well as altering the metabolic activity of the liver, driving inflammation and the oxidation of low-density lipoprotein (LDL).

Currently the role of COPD in the pathogenesis of atherosclerosis is under investigation, as both diseases share numerous common risk factors including a history of CS, persistent inflammation, a high oxidative stress burden, increased BP and unrestrained platelet activation (Figure 2) [10,60]. A recent study by Chandra et al. implicated a persistent air flow limitation and pulmonary endothelial dysfunction as strong independent predictors of atherosclerosis [61], whereas Topsakal et al. identified that patients with COPD have an increase in the intensity and severity of atherosclerotic disease, as more critical lesions were identified in patients with COPD when compared with those without the underlying disease. It was also evident that patients with COPD displayed worse morphological properties (enhanced calcification) within their stenotic lesions [62]. Topsakal et al. also speculated that both the chronic oxidative stress and inflammation associated with COPD may be what is driving coronary atherosclerosis within these patients.

The innate immune system has a crucial role in the development of atherosclerosis, with lipid-rich cells known as foam cells; derived from mononuclear phagocytes, being regarded as the key cell type responsible for the initial progression of disease due to their ability to uptake lipoproteins. It is the death of these foam cells that leads to the formation of the necrotic core within atherosclerotic plaques [63,64]. The accumulation of these mononuclear phagocytic cells drives local inflammation and the secretion of macrophage-derived pro-inflammatory mediators such as cytokines as well as promoting a complex adaptive immune response involving T lymphocytes. Vascular ECs have also been implicated in the early stages of atherosclerotic lesion formation due to their ability to secrete adhesion molecules including vascular cell adhesion molecule-1 (VCAM-1) upon exposure to inflammatory stimuli (cytokines) [65]. Chemokines also play a crucial role in the early stages of plaque development, chemokine (C–C motif) ligand 2 (CCL-2) and interferon-γ (IFNγ) induce T-cell recruitment and its associated inflammation [66,67] and macrophages are crucial to the development of the fibrous cap present on these plaques as a result of these cells containing the enzymes required for collagen production [68]. A study by Galis et al. showed that the expression of collagenases MMP-1, MMP-8 and MMP-13 is enhanced in atherosclerotic plaques, leading to thrombosis (favoring coagulation and platelet hyperactivation) and destabilization of these plaques, increasing the likelihood of further CVD complications such as ischemic stroke [69].

Under normal conditions, the vascular endothelium sustains blood vessel homeostasis, regulates cellular adhesion, promotes fibrinolysis (prevent blood clots) and maintains leukocyte aggregation [47]. Conversely, upon exposure to noxious stimuli such as CS, ECs lose their structural integrity leading to the secretion of pro-inflammatory mediators, enhanced secretion of VCAM-1 and a reduction in their anti-inflammatory ability, aiding in the recruitment of immune cells enhancing atherogenesis [70–73]. Hypoxia has also been shown to drive the development of CVD, with several studies showing that declined pulmonary function (FEV_1_ < 0.8) leads to an increased risk of ischemic heart disease and coronary artery risk in early adults [74,75].

A study by Fisk et al*.* recently highlighted various biomarkers which may be utilized in predicting cardiovascular risk in COPD patients [76]. The study utilized 458 COPD patients and 1657 non-COPD controls, where they were matched for age, sex and body mass index with the study finding that patients with COPD had an increase in aortic pulse-wave velocity, systemic inflammation as evidenced by elevated C-reactive protein levels, an increase in carotid artery thickness, arterial stiffness and subclinical atherosclerosis [76]. It has also been shown that both elevated levels of cardiac troponin and natriuretic peptides; two key prognostic cardiac biomarkers may be effective in the prediction of mortality in patients with COPD [77,78].

Although atherosclerosis and COPD are typically observed in the aging population, it is now understood that an increase in age is an independent risk factor of atherogenesis [79]. However biological aging causes reduced cellular proliferation, enhanced apoptosis and cellular senescence, which may explain why these two diseases are often observed together. Aging-related inflammation is also a common risk factor associated with COPD, CVD and atherosclerosis, therefore the role of systemic inflammation and oxidative stress in the manifestation of atherosclerosis needs to be elucidated further [80].

## Fueling the fire; inflammation and atherosclerosis

Comorbid CVD is currently the largest killer in patients with COPD, however the mechanisms underlying this disease are complex and largely unknown [60]. It has been shown that COPD and CVD share various common risk factors including CS, a sedentary lifestyle, low socioeconomic class and genetics [81]. Upon recruitment of immune cells and low-density lipoprotein (LDL) to the endothelium, there is further chemotactic signaling, inflammation and alterations to blood vessel function [82]. An important clinical biomarker is the presence of oxysterols; oxidized cholesterol, which has been observed in high concentrations in patients with atherosclerosis [83,84]. Atherosclerotic plaque formation generally occurs at vulnerable sites such as branch points where uniform blood flow is disturbed, allowing for low pressure points larger vessels such as the thoracic aorta and carotid arteries [85]. As outlined earlier impaired laminar flow causes oscillations and shear stress within the vessel, causing mechanical stress and mechanoreceptor activation (vascular endothelial growth factor receptor 2; VEGFR2 and platelet endothelial cell adhesion molecule-1; PCAM-1) by the vascular endothelium [86]. This subsequently promotes a spike in NO production, stimulating vascular relaxation and a reduction in BP/sheer stress. Endothelial activation then causes an increase in MMP signaling and immune activation. If the oscillating shear stress is persistent it causes mechanoreceptor activation and the inflammatory response is amplified, which implicates the nuclear factor κ-light-chain-enhancer of B cells (NF-κB) pathway driving the secretion of cellular adhesion molecules such as (VCAM-1 and PCAM-1), growth factors such as G-CSF and pro-inflammatory cytokines such as tumor necrosis factor α (TNF-α) and IL-6 [86]. NF-κB signaling in atherosclerosis highlights that this disease is largely due to chronic inflammation hence it being commonly seen in patients with pre-existing inflammatory conditions such as COPD. The recruitment of platelets, immune cells and cholesterol to the site of injury is key to the progression of these atherosclerotic plaques, however if NF-κB signaling is inhibited does this help reduce the likelihood of atherogenesis?

A study by Chiba et al. investigated the effectiveness of the NF-κB inhibitor dehydroxymethyl-epoxyquinomicin (DHMEQ) on atherosclerotic plaque formation and size in apolipoprotein E-knock out (ApoE^−/−^) mice [87]. The study concluded that ApoE^−/−^ mice treated with DHMEQ at both 4 and 16 weeks showed significantly lower atherosclerotic area, however their serum profiles for both triglyceride and cholesterol remained unchanged. Ariga et al. has also shown that DHMEQ also inhibits the activation of NF-κB signaling via TNF-α, preventing nuclear translocation of active NF-κB [88]. This highlights the clinical potential for novel therapeutic treatments that specifically target the pro-inflammatory mechanisms driving atherosclerosis.

Numerous studies have also shown that in the exhaled breath of both smokers and COPD patients there is an alteration of both NO and CO balance [89–92]. Montuschi et al. assessed if both exhaled CO and NO can be used as a biomarker of pulmonary oxidative stress *in vivo* [89]. Results from this study showed no clear correlation between exhaled CO levels and pulmonary function, however exhaled NO was elevated in ex-smokers with COPD, than in both healthy non-smokers and current smokers with COPD, with a negative correlation between NO levels and FEV_1_ [89]. Analysis of exhaled NO may be a useful clinically relevant biomarker of airway inflammation and oxidative stress in patients with COPD. Little is known about the effect of CS on systemic NO metabolism; however, studies have shown that exposure to CS causes a significant down-regulation in the levels of bioavailable l-arginine within the systemic vasculature [93–95]. A decrease in l-arginine concentration has adverse effects on the expression of eNOS, as l-arginine is necessary for the generation of NO through the activity of eNOS [10]. The down-regulation of l-arginine is primarily due to an increase in oxidative stress, with studies by Saisos et al. and Taddei et al. both highlighting the detrimental effect of both cigarette smoke (CS) and age-related oxidative stress on reduced NO, respectively [93,94]. Siasos et al., stated that l-arginine may be beneficial in the treatment of CVD as it improves overall endothelial function in healthy smokers [93]. This was investigated further through the oral administration of an l-arginine supplement. The results from this study showed that oral l-arginine was able to acutely improve endothelial function and the elastic properties within the brachial artery. With l-arginine being the substrate necessary for eNOS-dependent NO production as well as it improving endothelial function in patients susceptible to atherosclerosis [96], it is clear that l-arginine supplementation may be beneficial in the treatment of CVD, due to NO being crucial in the regulation of BP as well as potentially mediating thrombotic events and maintaining/preventing turbid blood flow surrounding atherosclerotic plaques.

## Platelet aggregation and atherothrombotic lesion formation and destabilization

Patients with atherosclerosis are often at risk of developing an atherothrombotic lesion, which can be defined as an atherosclerotic lesion that becomes disrupted due to a superimposed thrombus formation which is the leading cause of cardiovascular deaths and acute coronary syndromes (ACS) [97]. Typically, atherosclerosis progresses throughout the duration of a lifetime, starting in early childhood then progressing into adulthood, however this is largely asymptomatic. The development of this disease into more severe disease states such as atherothrombosis, can lead to complete vessel occlusions and subsequent ischemic stroke, coronary artery disease and peripheral arterial disease, thus effecting various tissue types and vascular regions [97]. Like atherosclerosis and lipid accumulation, one of the initial stages of atherothrombotic lesion formation is endothelial dysfunction, promoting systemic inflammation and immune cell recruitment. This immune cell recruitment can lead to structural modification of both the tunica media and the adventitial layers of the vessel wall [98]. Initially, atherosclerotic lesions can go undetected when a patient is subject to angiography, as a result of positive remodeling (vascular enlargement), thus the vascular lumen remains unaltered due to the combination of low levels of stenosis and compensatory enlargement of the lumen, thus the patient being asymptomatic [99].

**Figure 3:**
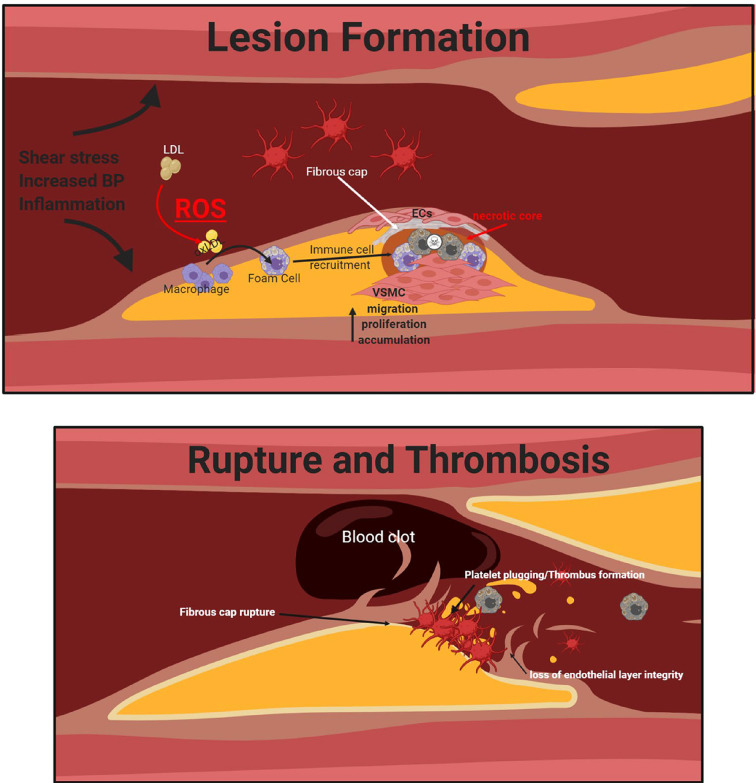
Atherogenesis, plaque rupture and thrombosis Enhanced shear stress, turbid blood flow, BP and inflammation mediate the formation of an atherosclerotic lesion. This then promotes the recruitment of immune cells, proliferation and accumulation of VSMCs and the oxidative modification of LDL to form harmful foam cells, a key component of the necrotic core of the lesion. Upon plaque rupture, a blood clot is formed, and thrombus formation occurs, making these patients highly susceptible to a further cardiovascular event.

It is the disruption of these atherosclerotic lesions that promotes the detrimental thrombotic process, triggering a multitude of effects inducing increased local shear blood flow, the release of apoptotic signals, increased monocyte and immune cell recruitment, secretion of tissue factors and thrombotic factors, all of which contribute to the development of the thrombus (Figure 3) [22,71,97,100,101]. Under normal conditions, the vascular endothelium plays a crucial role in maintaining platelet aggregation and the balance between pro- and anticoagulant factors through the secretion of prostacyclin (PGL2), NO, tissue fibrin inhibitors and regulating fibrinolysis. However, under pathological conditions, this endothelium-mediated homeostasis is lost allowing for the development of atherothrombosis [71]. Atherothrombotic plaques have a thin fibrous cap, which is a layer of connective tissue that covers the lipid core. A key constituent of the fibrous cap is the presence of inflammatory cells, particularly macrophages and foam cells. The rupture of the fibrous cap is often observed in acute MI and ischemic stroke [102]. Cap degradation is largely due to prolonged inflammation, degradation of the matrix by MMPs secreted by invading macrophages and a decline in matrix synthesis as a result of decreased cap smooth muscle cells (SMCs) [103]. The SMCs within the vasculature are known to produce connective tissue within the intimal layer of the blood vessel to increase plaque stability and reduce the likelihood of rupture, although the efficiency of this can be altered as a result of inflammation and immune cell recruitment [103–105]. Due to VSMCs having high levels of phenotypic plasticity which allows for adaptation to environmental stressors and changes, playing a crucial role in the regulation of atherosclerotic lesion formation and stability (Figure 3).

A study by Alexander et al. showed that through genetic deletion of IL-1, ApoE^−/−^ mice when fed a Western diet displayed increased plaque stability and reduced plaque remodeling, reducing the likelihood of plaque rupture and highlighting the clinical importance of targeted therapeutics which impair phenotypic alteration in VSMCs [106]. In the initial stages of the above study, it was hypothesized that through the deletion of IL-1, there would be a subsequent reduction in localized inflammation and immune cell recruitment [106]. The findings from this study suggest that by identifying the cell types within a lesion prior to treatment may confer better outcomes clinically, as plaque stability and lesion progression may be able to be regulated therapeutically reducing the likelihood of morbidity and mortality. Platelet-derived mediators such as platelet-derived growth factor BB (PDGF-BB) have been shown to induce phenotypic transformations in cultured SMCs [107]. In a preclinical model of atherosclerosis, Kozaki et al. used PDGF-receptor KO in ApoE^−/−^ mice, which yielded promising data, with a 67% reduction in the overall atherosclerotic lesion size as well as drastic reductions in SMC involvement and delayed fibrous cap formation [108].

Several studies have investigated the effect of genetic deletion of various inflammatory cytokines, with IL-1, IL-17 and TNF-α KO models [109–111]. Findings from these studies suggest that through genetic depletion of pro-inflammatory mediators there is a significant reduction in the atherosclerotic burden and severity. A review by Hasselbalch highlighted that chronic inflammation can trigger the progression of accelerated atherosclerosis and CVD in patients with underlying diseases such as cancer [110]. It was also mentioned that the use of both statins and interferon-α2 (IFN-α2); the inflammatory protein secreted by cells subject to a viral infection and inhibitory to cancer cell proliferation, has been shown to reduce thrombohemorrhagic complications as well as having strong anticancer properties [110]. These data suggest that modulation of inflammation may be crucial in reducing the overexuberant platelet activation observed in the development of atherothrombotic lesions. It has been well established that chronic inflammatory diseases such as rheumatoid arthritis, diabetes mellitus and COPD have been associated with premature atherosclerosis and the development of atherothrombosis [112–116], therefore the modulation of this inflammatory burden may be a potential novel therapeutic strategy for the treatment of atherosclerosis clinically.

## Current pharmacological treatments of atherosclerosis

### Statins, angiotensin-converting enzyme inhibitors and angiotensin receptor blockers

The most common treatments for atherosclerosis are cholesterol-lowering statins, angiotensin-converting enzyme (ACE) inhibitors and/or angiotensin receptor blockers (ARBs), which are often used in conjunction with one another. Statins have been shown to increase HDL, reduce LDL, triglyceride concentration and the incidence of CVD and coronary heart disease and its associated mortality [117,118]. Studies have been conducted into the potential adverse effects of statins, which concluded that there were no associated effects on outcomes including autoimmune diseases, thromboembolism or infection susceptibility, while showing promising benefits in cases of vascular disease [119]. Statin treatment is effective as it works through targeting hepatocytes, by irreversibly binding and inhibiting the activity of hydroxymethylglutaryl coenzyme A reductase inhibitor (HMG-CoA) reductase [120]. HMG-CoA reductase is responsible for the catalytic conversion of HMG-CoA into the cholesterol precursor mevalonic acid, thus causing a reduction in intracellular cholesterol and impaired secretion of triglyceride-rich lipoproteins. It is believed that statins may also have antioxidant properties, as they can prevent superoxide formation within ECs and attenuate the oxidation of LDL via preservation of antioxidant enzymes such as superoxide dismutase (SOD) [121,122].

Talbot et al. conducted a multiethnicity population-based perspective study consisting of 5280 participants of various ethnicities from multiple sites across the United States, investigating the role of lipid-related pathways and statin treatment in reducing mortality in atherosclerotic disease in a 5-year study [123]. The results from this study showed that statin treatment reduced the likelihood of coronary heart disease and CVD by 14 and 23%, respectively, while reducing the incidence of mortality by 18%. Statin treatment had no significant effect on altering HDL and triglyceride levels, however there was a significant reduction in overall LDL concentration, thus the reduction in morbidity and mortality observed in this study was attributed to modulation of LDL [123]. More recent evidence has emerged in regard to the clinical use of statins in COPD patients [124]. Lu et al. found that use of statins drastically reduced all-cause mortality, AECOPD and heart disease-associated mortality, while reducing CRP levels and pulmonary hypertension severity [124], therefore statin use may be beneficial in the treatment of both the pulmonary and cardiovascular manifestations associated with COPD/AECOPD.

Both ACE inhibitors and ARBs have been used extensively in the clinical treatment of hypertension and atherosclerosis. Ang II is formed by the conversion of angiotensinogen into angiotensin I by renin that is secreted by the kidney, which is further converted into Ang II by ACE. Ang II can then increase vasopressin production within the central nervous system as well as promote vasoconstriction within VSMCs, thus increasing BP. ACE-inhibitors act directly on the renin–angiotensin system by preventing the conversion of Ang I into Ang II as well as inhibit the degradation of bradykinin; a potent vasodilator that mediates NO release, lowering BP [125,126]. Due to the complex nature of cellular signaling, these drugs are often used in conjunction with other drugs as Ang II production can occur through alternate pathways which are unaffected by ACE inhibition, thus ARBs are generally better at inhibiting the effect of Ang II due to their ability to react specifically with Ang receptors [125]. ARBs, have a similar therapeutic effect, however they block the effect of Ang II through competitive inhibition of the Ang II receptor on the VSMC surface, preventing vasoconstriction [125,127]. An observational study by Kim et al. investigated the benefits of ACE inhibition/ARBs in combination with statin treatment in patient’s post-MI [128]. A total of 11706 patients were enrolled and separated into two groups; ACE-inhibitor + statin-treated and ARB + statin-treated groups, with endpoints described as mortality/major cardiac events, coronary revascularization or recurring MI. This study concluded that revascularization and MI frequency was unaltered among groups, however ACE-inhibitor + statin group had a significantly lower level of all-cause mortality than the ARB + statin group during a 2-year period [128], therefore combination treatment using ACE-inhibitors and statins is more beneficial in reducing long-term CVD-associated mortality.

The use of statins has also been associated with a reduction in acute exacerbation risk. A randomized clinical trial by Ingebrigsten et al. identified 5794 COPD patients in the Copenhagen General Population Study and measured their CRP levels, recorded exacerbations, hospital admissions and their use of oral corticosteroid treatment for 3 years post-study [129]. This study identified that statins in fact reduced the likelihood of acute exacerbations and CRP levels in COPD patients with comorbid CVD [129]. Use of these BP-lowering drugs has proven to be beneficial in reducing the incidence of cardiovascular mortality as well as the likelihood of atherosclerotic lesion formation while aiding in the treatment of other systemic manifestations and diseases such as COPD [130–133].

### ICS

Another common treatment for COPD is via ICS. These drugs have potent anti-inflammatory effects with a long duration of action within the airways than oral corticosteroids given their targeted delivery directly into the lungs [134]. However, there is concern surrounding potential off-target effects, due to the potential of these ICS being absorbed systemically, driving hypertension and glucose intolerance [135–137]. However, findings from a recent randomized controlled trial have shown that treatment with ICS significantly reduced all-cause mortality in COPD, and a reduction in the number of cardiovascular-associated deaths when compared with the non-ICS treated control group [138] The anti-inflammatory effects associated with ICS may be useful in the treatment of comorbid atherosclerosis in patients with COPD, as the use of corticosteroids can reduce systemic inflammation, improve cardiac function and reduce ventricular dysfunction [138,139]. In-line with this it has been shown that treatment with ICS in patients with COPD reduces the likelihood of coronary heart disease [140]. However, it has been suggested that corticosteroids can inhibit the activity of immune cell recruitment and adhesion to the vascular wall through negatively regulating the activity of VCAM-1, E-selectin and ICAM-1 [141,142], severely impacting the onset and development of atherosclerosis.

### Diuretics, β-adrenoceptor and calcium-channel blockers

Clinicians utilize an array of diuretics and calcium channel blockers to reduce BP and the shear stress exerted on the vascular wall. Thiazide diuretics (TDs) have been used extensively for the treatment of hypertension and are commonly used in patients with salt-resistant hypertension [143]. TDs work by reducing both diastolic and systolic BP, alleviating fluid congestion and the likelihood of cardiovascular morbidity. One downfall for the use of diuretics is that depletion of blood magnesium levels (hypomagnesemia) is often evident due to the increase in fluids expelled from the body. A randomized controlled clinical trial by Cunha et al. investigated endothelial function in patients with atherosclerosis that were treated with TD as well as exploring the effectiveness of oral magnesium supplementation [144]. The findings from this study showed that patients treated with TD + magnesium supplementation had a significantly greater decrease in both systolic and diastolic BP allowing for an increase in flow-mediated dilation, while having no adverse effects on plasma lipid or glucose content or worsened arterial stiffness. Conversely, patients treated with TD without magnesium supplementation showed much lower reductions in BP and surprisingly showed a significant increase in carotid-intima thickening, indicative of vascular remodeling due to elevated BP, and hence this group displaying impaired flow-mediated dilation. The study concluded that there was in fact a direct correlation between both intracellular magnesium concentration and flow-mediated dilation [144], thus magnesium supplementation provides improved endothelial function and drastically reduces the risk of atherosclerosis in at-risk hypertensive patients currently under TD treatment. If untreated, hypertension exerts excess shear force on the arterial wall, leading to arterial damage and causing the vasculature to be more susceptible plaque build-up and structural remodeling, hence atherosclerotic lesion formation. When coupled with other risk factors such as cigarette smoking, high cholesterol, or other underlying diseases such as COPD it then places these patients at an even higher risk of developing atherosclerosis and other cardiovascular comorbidities.

Hypertension has also been often implicated with alterations to ion transport, including the movement of both calcium and potassium, which promote vasoconstriction, VSMC proliferation and hypertrophy. Arterial remodeling is due to both increase in VSMC number (hyperplasia) and increased VSMC mass (hypertrophy), which occur as a result of enhanced BP [106,145]. The vascular ECs under normal conditions regulate the proliferation and growth of the underlying VSMCs, however under pathological conditions like that in hypertension and atherosclerosis, this regulation is lost due to endothelial damage and thus there is subsequent narrowing of the vascular lumen [145]. It is also important to highlight that hypertension promotes localized oxidative stress within the vascular wall and a subsequent reduction in eNOS expression [146] and that coupling this with exogenous ROS from exposure to pollutants such as CS places these patients at a heightened risk of CVD and worsened hypertension [147,148]. β-adrenoceptor blockers have also been used extensively in the treatment of hypertension and atherosclerosis. These drugs antagonize sympathetic activity within the heart while inhibiting the activity of ACE, reducing heart rate and BP, improving hemodynamic parameters such as cardiac output and stroke volume [149,150]. Quite commonly, β-adrenoceptor blockers are used post-cardiac and vascular surgery as they reduce the incidence of cardiac complications including angina and MI. However in the context of COPD, the prescription of β-adrenoceptor blockers is uncommon due to these drugs having deleterious effects on the pulmonary symptoms associated with this disease by blocking β_2_-adrenoreceptors and promoting bronchoconstriction [151], however there is compelling evidence to suggest that the administration of cardioselective β-adrenoceptor blockers is safe in patients with comorbid CVD and COPD [152–155]. This was shown in a study by Gestel et al. which evaluated 3371 patients who underwent major vascular surgery and further divided into subgroups of those with COPD and those without, based on spirometric analysis [152]. During this study, patients were subject to both a low dosage (<25% recommended maximal dosage) and an intensified therapeutic dosage (maximal specified dosage), with data showing that there is a dose-dependent reduction in the likelihood of cardiovascular mortality in patients with COPD following intensified dosage, but not at low dosage. These findings highlight that these cardioselective drugs may be beneficial in reducing short-term mortality in patients in patients with COPD, particularly post-surgery.

The use of calcium-channel blockers is also quite common in the treatment of atherosclerosis, as these drugs prevent calcium ion entry into the VSMCs, cardiac node tissue and cardiac myocytes through binding to L-type calcium channels. This blockade promotes vasodilation through reducing overall contractility by preventing calcium entry into VSMCs as well as promoting a decline in atrioventricular signaling ultimately reducing heart rate and BP [156]. These compounds exert their effects largely in arterial resistance vessels while having minor effects in the venous capacitance vessels [156,157], hence their use in atherosclerosis, in particular within branch points of the carotid and thoracic aorta. Not only are these drugs effective in the treatment of hypertension due to their ability to reduce BP, they are also useful in the treatment of atherosclerosis and have been shown to be effective preclinically in the inhibition of atherosclerotic lesion formation due to their ability to reduce the accumulation of lipids, matrix protein and calcium ions in the vascular wall [158,159]. Recently a study by Nezu et al. investigated the clinical effectiveness of Cilnidipine; an L/N-type Ca^2+^-channel blocker (blocks both L-type and N-type sympathetic nerve channels) in a group of post-stroke hypertensive patients with carotid atherosclerosis [160]. Throughout the study, analysis of both the intima-media thickness (IMT) and interadventitial diameter (IAD) was completed using ultrasonography. Analysis of both IMT and IAD was completed at the beginning of cilnidipine treatment and again 12 months later, with the findings from this study showing that the use of this calcium channel blocker significantly promoted carotid IMT regression, however displaying greater effectiveness in patients with more severe vascular remodeling (thicker arterial walls), as well as reducing the overall IAD in all patients [160]. This study suggests that the use of L/N-type calcium-channel blockers allow for the regression of carotid atherosclerosis and potentially reducing the likelihood of mortality in these patients.

### Antiplatelet drugs

COPD has been linked to severe increases in both platelet activation and atherothrombosis [161,162]. It has also been shown in a population-based cohort study that patients with underlying COPD are at a heightened risk of plaque destabilization and rupture due to increased arterial wall thickening and remodeling, thus COPD being an independent predictor of vulnerable plaques [163]. An early study by Ponicke et al. showed that the enhanced platelet activation observed in COPD patients is often associated with hypoxia and hemodynamic stress as a direct result of thrombus formation, aggregation, hypoxemia and heightened expression of cyclooxygenase-1 (COX-1) [164]; an enzyme responsible for platelet aggregation and renal regulation contributing to Ang II-induced hypertension [165]. Ekström et al. explored the time-dependent effects of cardiovascular drugs in patients with severe COPD; who are on long-term oxygen therapy [166]. The most common prescribed anti-platelet treatment is aspirin, of which has shown to cause a significant decrease in mortality within this high-risk cohort, whereas β-blockers were shown to decrease patient survival. Platelet activation is complex and requires COX-1 to convert arachidonic acid into prostaglandin PGH_2_ which is catalytically converted into thromboxane A_2_ (TXA_2_) through the activity of thromboxane synthase, promoting vasoconstriction and platelet activation and aggregation [10,167]. However, aspirin is an irreversible inhibitor of COX-1 as well promoting down-regulation in the activity of prostaglandins and TXA_2_ [167], therefore having a significant impact on platelet aggregation and reducing the likelihood of atherothrombotic events in at risk patients. The increased survival resulting from anti-platelet treatment is believed to be due to modulation of platelet activation and thus a systemic anti-thrombotic effect [161,166], therefore aspirin treatment may be beneficial in the treatment of comorbid atherosclerosis in patients with established COPD.

## Novel therepeutic approaches to treat atherosclerosis in COPD

The development of novel therapeutics is a major focus of modern research. In the context of COPD there is increasing interest into modulation of key inflammatory and oxidative stress pathways which have been linked to increased disease severity, extrapulmonary comorbidities and mortality [4,5,168–176]. It is believed that by increasing anti-inflammatory and antioxidant activity there may be a significant reduction in both systemic and pulmonary inflammation and oxidative stress, aiding in the preservation of lung function and reduce the instance and severity of comorbid diseases such as atherosclerosis. It has been established that the expression of inflammatory mediators such as TNF-α is significantly enhanced upon exposure to noxious stimuli such as CS [28,29]. Inflammatory mediators, in particular TNF-α, has been shown to significantly decrease the expression of glutathione; a key antioxidant, in the airways leading to exacerbated tissue damage to the lung parenchyma [177,178].

A study by Funamoto et al. investigated the anti-inflammatory effect of curcumin (Theracurcumin®) on patients with COPD and its ability to reduce oxidized α1-antitrypsin-LDL (AT-LDL) [179]. It has been shown that AT-LDL is found at sites of atherosclerotic lesion formation, and the presence of this AT-LDL within blood is indicative of foam cell activation within the lesion [180,181]. Under pro-inflammatory and oxidative conditions serum amyloid A (SAA) binds to LDL rather than HDL promoting further localized inflammation as a result of enhanced immune cell recruitment to the lesion site [182]. Findings from this study showed no significant alteration in LDL-cholesterol and BP, however there was a drastic reduction in AT-LDL levels following Theracurcumin® treatment, thus treatment with this novel therapeutic is beneficial in reducing atherosclerotic AT-LDL in humans and may reduce the likelihood of atherosclerotic events in patients with COPD. There is also compelling evidence to suggest that this compound may be beneficial in the treatment of the pulmonary manifestations of COPD. Curcumin was shown to act as both an antioxidant through reducing oxidative stress as well as exhibiting anti-inflammatory properties through its ability to inhibit the activity of NFκB following ischemia reperfusion injury *in vivo* by Fan et al. [183]. This study also showed significant reductions in both bronchoalveolar lavage fluid (BALF) and serum concentrations of IL-6 and ICAM-1, enhanced SOD expression and a reduction in myeloperoxidase activity and NF-κB signaling, highlighting a potential role for curcumin in the treatment of not only CVD but also in the modulation of chronic lung disease such as COPD.

There is increasing interest into the development of drugs that reduce oxidative stress in COPD as it is a key driver inflammation and disease progression [174,184]. Studies have been conducted in the past to investigate if modulation of ROS is of any clinical benefit in the treatment or prevention of atherosclerosis, however these studies utilized naturally occurring vitamins such as vitamins C and E, which have been shown to improve vascular endothelial function although showed very little efficacy in the treatment of atherosclerosis [185,186]. Interestingly, a recent study investigating the effects of vitamin C treatment (both prophylactically and as a treatment in established disease) in CS-induced emphysema using senescence marker protein-30 knockout mice, which cannot synthesize vitamin C, showed that vitamin C administration caused a significant reduction in oxidative stress, improved VEGF expression within the lungs and increased collagen synthesis in both groups [187]. This finding suggests that vitamin C supplementation prevents the onset of emphysema and aids in revering emphysematous damage in established disease [187]. Oxidative stress plays a powerful role in the development of COPD and its comorbidities, however during atherogenesis the use of antioxidants, those that are naturally occurring yield poor efficacy. This highlights the need for further medical research into antioxidant drugs such as NOX inhibitors (apocynin), SOD mimetics (AEOL 10150) and glutathione peroxidase(GPx) mimetics (ebselen) which offer greater potency and antioxidant activity. There is a current phase 4 clinical trial investigating the effect of selenium supplementation in the treatment of COPD through the modulation of blood antioxidant levels, based on a study by Espinola-Klien et al., which demonstrated that selenium supplementation causes an increase in GPx-1 activity and thus reducing the instance of cardiovascular events and mortality [188]. Ebselen, which is also a selenium-based compound, has been used by our laboratory previously, yielding exciting data due to its ability to reduce pulmonary inflammation and immune cell infiltration following CS exposure [28]. Our laboratory has also shown that ebselen treatment is able to completely prevent vascular dysfunction in both stable and viral exacerbated COPD as well as preserving skeletal muscle mass and reducing immune cell recruitment into the lungs *in vivo* [2,31].

## Conclusion

Both COPD and CVD share a wide array of common etiological factors that aid in disease progression, making CVD the largest killer of COPD patients globally. Currently, COPD is the third leading cause of deaths, costing the economy a staggering €82 billion annually [189]. There are limited treatments available that can effectively treat both COPD and CVD simultaneously, with mounting evidence suggesting the role of both inflammation and oxidative stress as drivers of COPD and atherosclerosis via platelet activation and the oxidation of LDL, leading to worsened clinical outcomes in these patients. We have previously shown that through antioxidant treatment we were able to significantly reduce lung inflammation and preserve vascular endothelial function in a murine model of COPD [2]. With endothelial dysfunction, oxidative stress and inflammation being hallmark characteristics in the development of atherosclerosis, antioxidant treatments may ultimately reduce atherogenesis in patients with chronic respiratory illnesses via reduced oxidation of LDL and lung inflammation as well as reducing the vascular oxidative stress burden. With a severe lack of research around atherosclerosis in COPD, it is crucial that we understand the complex inflammatory and oxidative stress mechanisms, allowing the development of novel life-saving therapeutics that can treat both the pulmonary and systemic aspects of this debilitating disease.

## Abbreviations

ACE: angiotensin-converting enzyme
AECOPD: acute exacerbations of chronic obstructive pulmonary disease
Ang II: angiotensin II
ApoE^−/−^: apolipoprotein E-knock out
ARB: angiotensin receptor blockers
AT-LDL: α1-antitrypsin-LDL
BP: blood pressure
COPD: chronic obstructive pulmonary disease
COX-1: cyclooxygenase-1
CS: cigarette smoke
CVD: cardiovascular disease
DAG: diacylglycerol
DHMEQ: dehydroxymethyl-epoxyquinomicin
EC: endothelial cell
eNOS: endothelial nitric oxide synthase
FEV_1_: forced expiratory volume in 1 s
GPx: glutathione peroxidase
HMG-CoA: hydroxymethylglutaryl coenzyme A reductase inhibitor
IAD: interadventitial diameter
ICS: inhaled corticosteroids
IL: interleukin
LDL: low-density lipoprotein
MLCP: myosin light chain phosphatase
MI: myocardial infarction
MMP: matrix metalloproteinase
NF-κB: nuclear factor κ-light-chain-enhancer of B cells
NO: nitric oxide
PC: phosphatidylcholine
PCAM-1: platelet endothelial cell adhesion molecule-1
PVAT: perivascular adipose tissue
PVD: peripheral vascular disease
ROS: reactive oxygen species
sbN_2_-test: single-breath N_2_ test
SMC: smooth muscle cell
SOD: superoxide dismutase
TD: thiazide diuretic
TNF-α: tumor necrosis factor α
TXA_2_: thromboxane A_2_
VCAM-1: vascular cell adhesion molecule-1
VSMC: vascular smooth muscle cell
